# Effective lane detection on complex roads with convolutional attention mechanism in autonomous vehicles

**DOI:** 10.1038/s41598-024-70116-z

**Published:** 2024-08-19

**Authors:** Vinay Maddiralla, Sumathy Subramanian

**Affiliations:** School of Computer Science Engineering and Information Systems, Vellore Institute of Technology, Vellore, 632014 India

**Keywords:** Attention mechanism, Object detection, Sharpening filter, Autonomous vehicles, Convolutional neural network, Lane detection, Semantic segmentation, Engineering, Mathematics and computing, Computer science, Information technology

## Abstract

Autonomous Vehicles (AV’s) have achieved more popularity in vehicular technology in recent years. For the development of secure and safe driving, these AV’s help to reduce the uncertainties such as crashes, heavy traffic, pedestrian behaviours, random objects, lane detection, different types of roads and their surrounding environments. In AV’s, Lane Detection is one of the most important aspects which helps in lane holding guidance and lane departure warning. From Literature, it is observed that existing deep learning models perform better on well maintained roads and in favourable weather conditions. However, performance in extreme weather conditions and curvy roads need focus. The proposed work focuses on presenting an accurate lane detection approach on poor roads, particularly those with curves, broken lanes, or no lane markings and extreme weather conditions. Lane Detection with Convolutional Attention Mechanism (LD-CAM) model is proposed to achieve this outcome. The proposed method comprises an encoder, an enhanced convolution block attention module (E-CBAM), and a decoder. The encoder unit extracts the input image features, while the E-CBAM focuses on quality of feature maps in input images extracted from the encoder, and the decoder provides output without loss of any information in the original image. The work is carried out using the distinct data from three datasets called Tusimple for different weather condition images, Curve Lanes for different curve lanes images and Cracks and Potholes for damaged road images. The proposed model trained using these datasets showcased an improved performance attaining an Accuracy of 97.90%, Precision of 98.92%, F1-Score of 97.90%, IoU of 98.50% and Dice Co-efficient as 98.80% on both structured and defective roads in extreme weather conditions.

## Introduction

As reported by the World Health Organization (WHO), over 1.19 million people have lost their lives and almost half of the million people under 5 to 29 years are the greatest cause of death due to injuries from road crashes^1^. The level of curiosity has risen substantially towards the development of automated driving capabilities and, ultimately, completely automated cars to reduce the traffic and accidents. A crucial component is the safety of these automated activities^2^. A critical step in autonomous driving recognizes traffic patterns around cars. In congested traffic conditions, it is crucial for self-driving systems to be able to recognize road markings, especially lane boundaries. This information is vital for modules involved in path planning and decision-making. Human drivers primarily rely on their eyesight while operating automobiles and market prices of vision sensors are inexpensive^3^. Recent computer vision and machine learning developments have strengthened vision-based techniques^4^. As a result, the most popular approach to recognizing lanes is the vision-based lane detection algorithm. Camera-based lane identification^5^ is crucial for environmental awareness, making it possible for the vehicles to navigate inside the lane. The majority of lane departure alert and lane-holding systems are built on this principle. Conventional vision-based lane-detection algorithms typically function in a four-step process, involves recognising lines and fitting, feature extraction, image pre-processing, and post-processing^6^. These approaches rely on low-level, manually generated elements including colour, gradient, and ridge patterns^7^. The four-step process often uses conventional computer vision techniques including inverse perspective mapping^8^,^9^, the Hough transform^10^, Gaussian filters^11^, and random sample consensus^12^,^13^. These algorithms do well in locating lane markers on roads with well-lit environments but perform poorly when lane borders are hidden when the trials are conducted in poor weather^14^. This is due to the fact that lane borders fluctuate in various traffic conditions^15^, but hand-crafted features are unable to simultaneously satisfy the needs of several scenes^16^. As a result, traditional approaches have two drawbacks: (a) a single picture is constantly needed for recognition outcomes; and (b) The individually produced features can be difficult to maintain and can occasionally not be suitable. As a result, the detection accuracy is not quite robust.

Over the past 10 years, advances in algorithms for deep learning and processing capability have led to the development of many efficient deep neural network-based lane detection techniques^17^,^18^. In certain algorithms, CNN was used as a method for extracting features to determine lane borders, and it consistently demonstrated good accuracy^19^,^20^. Despite the benefits, the majority of CNN techniques are trained on data from excellent road conditions. Extreme road conditions that can adversely influence the functioning of the model and the autonomous car have not yet been taken into account by researchers. But most emerging nations don’t have adequate infrastructure planning. Financial constraints make it difficult to complete the routine upkeep of transmission channels and the creation of ideal transportation systems for the deployment of current lane detecting networks to introduce autonomous cars in a timely manner^21^. As a result, this has become a problem for low-income nations, as there aren’t many studies that specifically address these problems. And it is computationally demanding, nevertheless, making it challenging to implement them in autonomous systems with constrained computer power. The main motivation of this work is to precisely recognize the lane markings in challenging roads with curves, faulty pavements and handling extreme weather conditions in autonomous vehicles.

The following contribution of this work is briefed which will encourage research in autonomous driving:

- With E-CBAM model, all the lanes in the road image is detected accurately without any loss of original image resolution on filtering the non-lane objects.
- To increase the training performance in deep neural network a skip connection is established in residual block and attentive normalization is used to normalize the complex features in terms of representation learning and improved performance.
- The proposed model is applied to extreme weather conditions that includes shadows, curves, rain, broken and no lane markings that results to low false positives and good accuracy performance that can ensure the safety for autonomous driving vehicles.

The paper is organised as follows: In Section "Literature survey", a discussion of the most recent lane recognition methods is presented. An elaborate summary of the research technique is given in Section "Proposed LD-CAM Architecture for Lane Detection". In Section "Encoder unit", "Enhanced Convolutional Block Attention Module (E- CBAM)" and "Decoder unit", scenarios, the research procedure and assessment findings of lane detection are presented and analysed. Last section covers the Experiments and Results, followed by Conclusion.

## Literature survey

According to the literature, computer vision-based algorithms have been frequently used to address lane detecting challenges. These machine vision-based approaches are broadly classified as classical and Deep learning based.

Yao et al.^22^suggested an enhanced deep neural network (DNN) for attention, which consists of two branches operating at various resolutions and is a lightweight semantic segmentation architecture designed for effective computation in less memory. The suggested network computes dense feature maps for prediction tasks at low resolution in global context. Multiple attention techniques are implemented based on the properties of differential feature resolution qualities. However, the accuracy of the model is not improved. Tabelini et al.^23^provided an innovative approach to lane recognition using deep polynomial regression to produce polynomials that represent each lane marking in the picture from an image captured by a forward-looking camera installed in a vehicle. However, the accuracy of the model has dropped drastically. The model might not have been able to learn this circumstance from less number of samples available since the pictures in those scenarios have a significantly different structure. Zhang et al.^24^enhanced existing semantic segmentation approaches for lane detection and provided a DC-VGG-SAD network (VGG: visual geometry group) that uses dilated convolution (DC) to lower network complexity and guarantee detection accuracy. Moreover, including self-attention distillation (SAD) accelerates the transfer of information. Zhang et al.^25^suggested a spatiotemporal network with double Convolutional Gated Recurrent Units (ConvGRUs) to handle lane detection in difficult scenarios. The locations and functionality of the two ConvGRUs in the network are distinct, although with identical architecture. Moreover, this work detects more information than the marked length leading’s to more false positive results. Lu et al.^26^proposed a method known as Scene Understanding Physics-Enhanced Real-time (SUPER) for real-time lane recognition. The suggested approach is divided into two primary modules: a multi-lane parameter optimisation module for lane inference that is strengthened by physics, and a hierarchical semantic segmentation network acting as the scene feature extractor. Identifying complicated lane scenarios is the concern. Lee et al.^27^provided a lightweight UNet for end-to-end learning of path prediction (PP) and lane identification in autonomous driving that uses depthwise separable convolutions (DSUNet). Additionally, convolutional neural network (CNN) incorporated with PP to create a simulation model (CNN-PP) that can be used to evaluate the qualitative, quantitative, and dynamic performance of CNN in a host agent car that is autonomously driving alongside other agents in real time. This research lacks real time implementation when the host is travelling alongside with other agents. Liu et al.^28^provided a reliable lane recognition model for complicated driving scenarios by combining contextual driving information with vertical spatial characteristics. The suggested model can detect unclear and obstructed lane lines more robustly with the two developed blocks, the feature merging block and the information exchange block that makes better use of contextual information and vertical spatial characteristics. However, it does not provide the computational analysis for real time applications and resource constrained systems. Jinfu Chen et al.^29^ proposed the AIdetectorX-SP model, which aims to enhance VD capabilities and efficiently recover vulnerability logic aspects by including a Temporal Convolutional Network (TCN) and Self-attention Mechanism (SaM). Overall aim was to create an automatic feature design detection model for software vulnerabilities with improving detection accuracy and lowering false positive and false negative rates. Yassine Oukdach et al.^30^ worked on automatic identification of gastrointestinal disorders using video capsule endoscopy (VCE) pictures by fusion of deep learning model with fusion transformers and CNN with attention mechanism to improve the classification performance in VCE images. Deepak Kumar Dewangan et al.^31^ carried experiments on detection of speed bumps by applying vision-based convolutional neural network model and SBDNet for autonomous vehicle system. Also, it draws attention to the experiment’s learning rate parameter and its analysis, highlighting how crucial it is to the speed bump detection network’s functionality. Deepak Kumar Dewangan et al.^32^ worked on detection of road conditions in different scenarios such as clear, fog, snow, rain and night roads. In order to segment road regions, the study presents a unique model named DSLnO + FCN, which is based on the VGG-net architecture and takes location prior data, RGB channel, and semantic form into account. Mallikarjun Anandhalli et al.^33^ Tresearched to overcome the shortcomings of current techniques by creating a revolutionary vehicle recognition algorithm that can recognize vehicles in a variety of climatic conditions and complex surroundings. They also focussed to localize vehicles using corner sites and group those places to increase the precision of traffic surveillance systems, boosting the overall traffic management and monitoring performance. Mallikarjun Anandhalli et al.^34^ carried out work on five essential steps such as corner detection, modeling, tracking and grouping for vehicle recognition. Their proposed algorithms ability is to correlate closely adjacent corner points within vehicular zones, based on Euclidean distance which helps to improve the accuracy of vehicle recognition and tracking. Mallikarjun Anandhalli et al.^35^ research focuses on vehicle detection and measuring the vehicle speed which voilates the traffic rules. Also, mapping the geometric plane of the physical environment onto the image plane allows for the calibration of a vehicle’s distance covered in both the image plane and the real world, which helps with accurate speed calculation.

According to the literature, several CNN-based techniques have previously been proposed to enhance the result for recognizing lanes in a variety of situations as shown in Table 1. Because to a paucity of training data on terrible roads, however, relatively few research has concentrated on creating simple CNN-based techniques that can predict lanes properly in bad weather and on poorly constructed roads. Moreover, while applying the attention technique, the edge information has not been correctly obtained. The other restriction when employing the attention mechanism is the need for more wasted computational resources. So, in this research, a unique CNN-based lane recognition model is created using a mixed dataset and evaluated in a variety of weather and road conditions.

**Table 1:** Comparision table for Literature interms of methods, Limitations and Performance measures.

Reference	Methodology	Observation	Limitation	Performance Measures
Yao et al.^22^	Deep Neural Network, Semantic Segmentation Architecture	Carried work on lane detec-tion tasks in different cate- gories with improved accu- racy and memory overload problems	Need more discussion on scalability problems	Accuracy False Positive (FP) False Negative (FN) FPS Parameter(M)
Tabelini et al.^23^	PolyLaneNet, Linear Regression	Captured picture from a forward looking camera to detect lane lines	Need to explore on segmentation tasks	Accuracy FP FN
Zhang et al.^24^	DC-VGG- SAD network	Improving lane detection approach for autonomous vehicles with fast processing speed	Struggles in accurate lane detection in complex condition such as night time and no- lane conditions	Accuracy False Positive False Negative Precision Recall F1 Score
Zhang et al.^25^	Double Convolutional Gated Recur rent Units (ConvGRUs)	Efficiently extract temporal and geographical data for precise lane detection, partic- ularly in difficult situations involving occlusions, curves, intersections	Conditions like fog, snow and sandstorms can be focussed	Accuracy Precision Recall F1-Score
Lu et al.^26^	Scene Understanding Physics-Enhanced Real-time (SUPER) Algorithm	Focused on scene understanding to detect lane lines using monocular cameras	Furthur focus on unparalled lanes such as merging two lanes and lane split condi- tions	Fmax Precision Recall FPR
Lee et al.^27^	DSUNet, CNN-PP	Experiments done on lane detection , road recognition and path prediction in AV	Model Robustness lacks in different road conditions	Accuracy Precision Recall F1 Score
Liu et al.^28^	Encoder – Decoder Architecture	Contextual and geographical features are highlighted increasing the accuracy of lane detection	Needs focus on urban conditions with dense traffic	Accuracy Precision Recall F1 Score
JinfuChen et al.^29^	CNN, Temporal Convolutional Network (TCN),Self- attention Mechanism (SaM)	Able to effectively extract vulnerability logic aspects from source code, improving the accuracy of VD	Further focus on effectively depict source code portions in order to get more data	Accuracy False Positive Rate (FPR) False Negative Rate (FNR) Precision Recall F1 Score
Yassine Ouk-dach et al.^30^	CNN, Attention Mechanism	Disease detection in video capsule endoscopy (VCE) images	Facing difficulties in finding anomalies in small images, which may affect in real world	Accuracy Precision Recall F1 Score
Deepak KumarDe- wangan et al.^31^	CNNbased SBNet	Experiments on marked and unmarked bump for AV’s	Limited discussion on challenging conditions like low illuminations and deprived roads	Accuracy Sensitivity F-Measure
Deepak KumarDe- wangan et al.^32^	DSLnOand FCN Algorithm	Accurate detection of road regions with RGB images	Futhur focus on Real world scenarios	Accuracy Precision F1-Score
Mallikarjun Anandhalli et al.^33^	Lucas- Kanade algorithm	Detect and localize vehicles based on corner points in different weather conditions	Need to address the problem where, when two vehicles approach each other too closely, they are perceived as one	Accuracy Correctness Completeness Quality
Mallikarjun Anandhalli et al.^34^	Corner detection algorithm	Determine the corner regions within the vehicle areas	Additional progress in lowering false positives, particularly in occlusions or complicated backdrops	Precision Recall Accuracy
Mallikarjun Anandhalli et al.^35^	Kalmanfilters, Image processing Vehicle	Vehicle speed, tracking and motion detection	Furthur focus on different scenarios	Sensitivity Specificity Accuracy

### Ethics approval

The work doesn’t require any approval from ethics committee.

## Proposed LD-CAM Architecture for Lane Detection

Lane detection accuracy is crucial for autonomous vehicles. However, traditional methods require hand-crafted features and post-processing procedures. Deep Learning models have been proposed for pixel-level lane segmentation, which focus on high accuracy on structured roads and good weather conditions, neglecting unstructured roads like cracked lane lines and curvy ones, and often have complicated architectures.

An LD-CAM architecture for lane detection in unstructured roads is proposed in this work. The preliminary pre-processing module focuses to reduce the size of the input image thereby reducing the memory and applies linear interpolation to estimate the missing data in the dataset. This helps to speed up the process. Further, the RGB colour image is transformed into gray-scale image.

**Figure 1:**
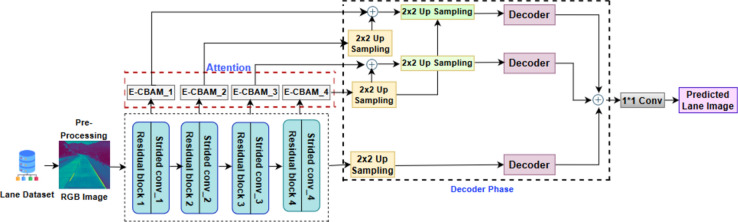
LD-CAM Architecture.

The pre-processed image is then fed to LD-CAM architecture which consists of an encoder, Enhanced Convolutional Block Attention modules (E-CBAM), and a decoder. The encoder extracts essential features from the pre-processed image and builds low-level (edges, corners, textures) and high-level (lane boundaries, lane markings, lane curvature, lane transitions, and road geometry) feature maps. The features created by the encoder section is transmitted through the attention module block which focuses on the main features such as lane marking, lane transitions and lane curvature to extract these features on road region. The decoder module reconstructs the feature maps collected from the unique attention module to provide the predicted images with the exact resolution as the input images. Figure 1 depicts the general framework of proposed LD-CAM Architecture. In the next sections, the architecture of every component of the model is briefly examined.

## Encoder unit

**Figure 2:**
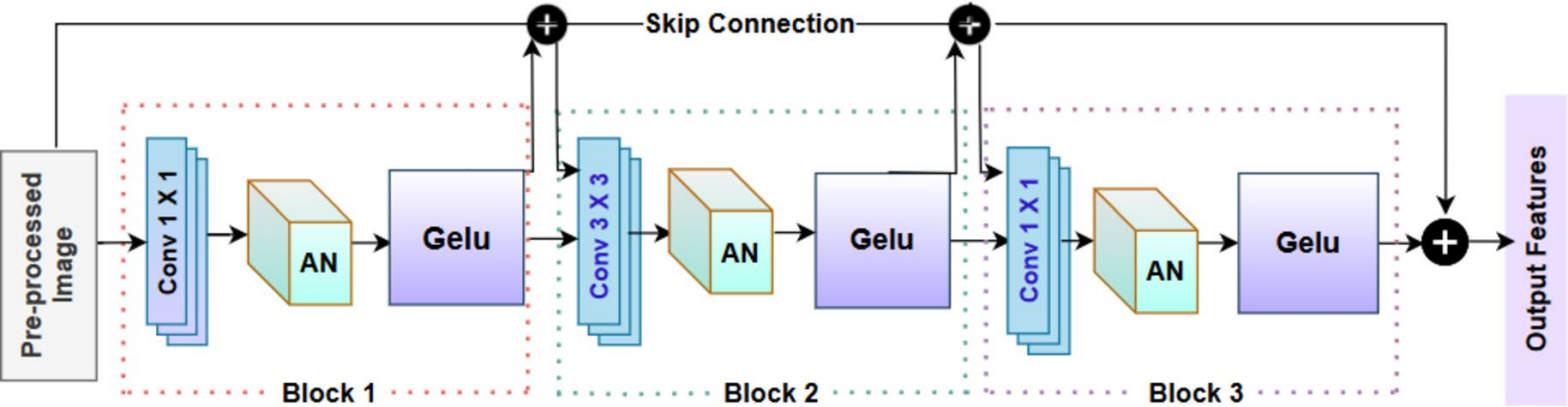
Residual block.

The encoder module consists of four residual blocks and four strided convolution layers. Each residual block of the encoder module consists of three convolutional layers, three normalisation layers, and three activation functions which assures reliable feature extraction and transformation. The four strided convolution layers of the encoder module takes care of down sampling and concentrates on the pre-processed image features with minimal loss. The input feature maps are normalised using attentive normalisation (AN), which is an essential step in stabilising and improving the training procedure. Gaussian Error Linear Unit (GELU) is used as the activation function for convolutional layers, which combines the positive aspects of Rectified Linear Units (ReLUs) with dropout. The issue with ReLU is that, when the input lane image of a neuron in the network has a negative value, it is considered as zero. Hence, to overcome this, the proposed work has opted GELU activation function for smoother flow and to avoid overfitting problems. The construction of the residual blocks is displayed in Fig. 2.

Using a filter size of 1 × 1, the first convolutional layer in the residual block produces feature maps with a dimension of ’n’, which is the number of feature maps generated by this convolutional layer in the residual block. The 1 × 1 convolution acts as a pointwise convolution in this block and enables the model to modify the channel-wise data and represents intricate correlations between various features. The main advantage of this filter is that it focuses on main features and makes the network easier to handle. Next, attentive normalization adaptively modifies normalization parameters such as scaling and resizing which concentrates on pertinent features such as lanes. The network can dynamically scale and change the normalization based on the significance of various traits with the help of attention mechanism. This results in more efficient learning and improved model performance. The Gaussian Error Linear Unit (GELU) is used as an activation function to improve the performance of the deep learning model without falling into vanishing gradient problems. The activation function of GELU is as follows in Eq. (1);

1 $$GELU\left(l\right)=0.5l(1+tanh(\sqrt{\frac{2}{\pi }}(l+0.044715{l}^{3})$$

where, l is the input to the GELU function, tanh is the hyperbolic tangent function, $\sqrt{\frac{2}{{\varvec{\pi}}}}$** ,** 0.044715 are constant factor. A smooth, differentiable function GELU is useful for gradient-based optimization and training. Because of its zero-centeredness, the vanishing gradient problem is lessened and neural network convergence is aided^36^. In contrast to the hyperbolic or sigmoid tangent functions, GELU does not reach saturation at higher input values. Further, a 3 × 3 filter size namely the second convolutional layer is used to transfer the extracted features from the GELU. The 3 × 3 convolutional layer captures hierarchical characteristics in the input data, unlike the first 1 × 1 convolution, which is mainly concerned with altering channel-wise information and collecting complicated relationships. The second convolutional layer allows the model to capture fine-grained features and local relationships in the data by using the 3 × 3 filter. The Residual block identifies and considers patterns which encompasses many pixels due to its higher filter size, thereby enhancing the recognition of deeper patterns in the input. To achieve a balance between collecting local and global characteristics, it is typical to employ many convolutional layers in a residual block with different filter sizes. Within the residual block, the last convolutional layer uses a 1 × 1 filter with a size of ’n*2.’ This method of applying 1 × 1 convolutions helps control the computational complexity while preserving the ability to detect complicated patterns in the data.

The skip connection is proposed to tackle the problem of disappearing gradients. The output from the residual block 1 that comes after the GELU layer is integrated and sent as the input to the second residual block and so on through this skip connection. For the gradients during backpropagation, this creates a straight channel for the skip connection to mitigate the vanishing gradient problem, ensuring that the model can efficiently learn from the input data. The skip connection effectively functions as a shortcut, allowing the gradient flow through the deep learning model to be smoother. Once the output from residual block has been passed through the strided convolution layer, it is down-sampled by a factor of two times the size of the input feature map. At the final encoder block, the downsampled and altered representation of the input data is reflected in a feature map with dimensions of H/16 × W/16 × C. Finally, it extracts the low level and high level features and fed into the E-CBAM module.

## Enhanced convolutional block attention module (E-CBAM)

**Figure 3:**
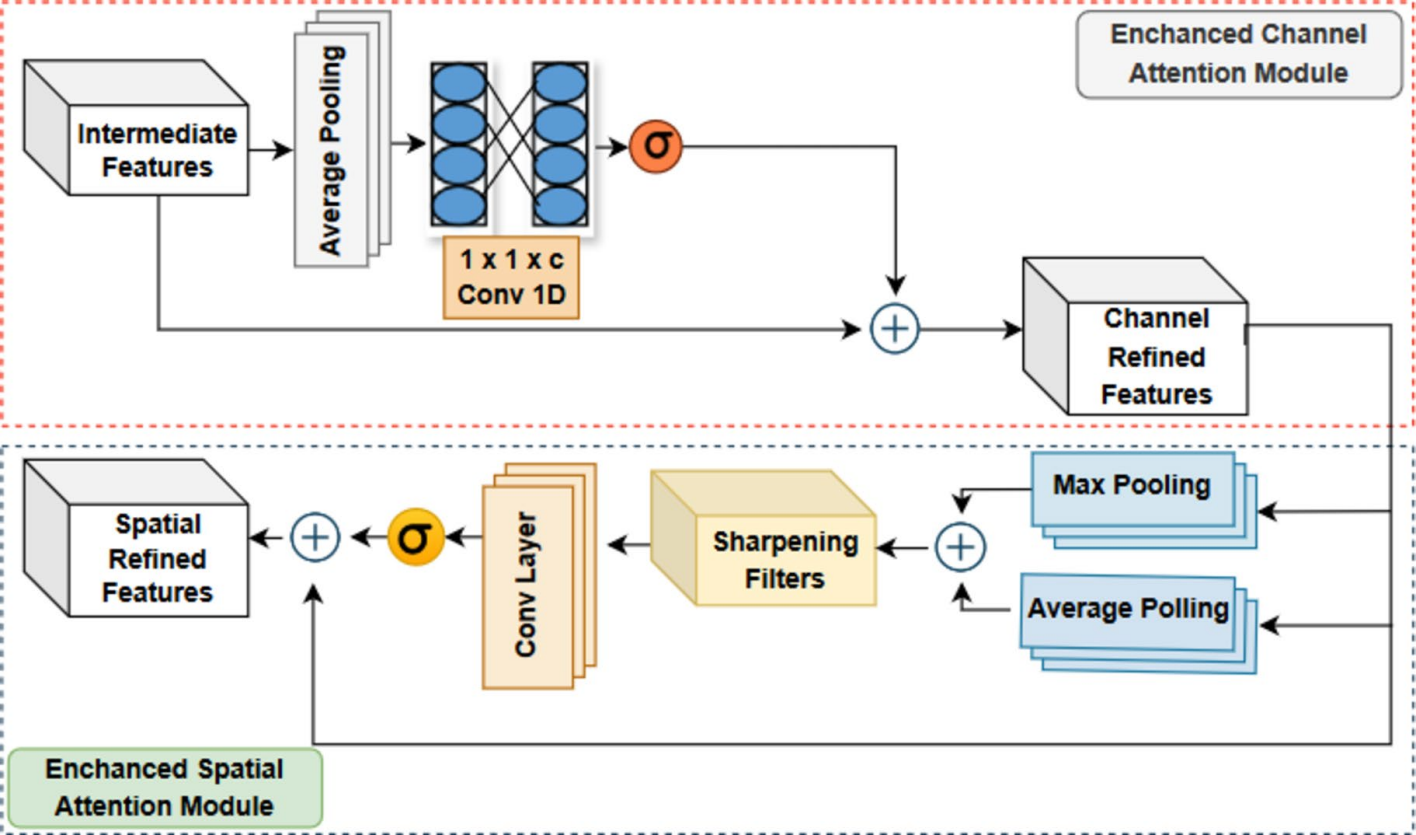
E-CBAM Architecture.

A critical processing step is carried out by the refined activation maps through Enhanced-Convolutional Block Attention Modules (E-CBAMs), which preserve the same size as the intermediate activation maps acquired from the encoder stages. These intermediate activation maps come from four phases, each of which represents a particular stage of the model’s feature extraction process. To improve the characteristics of the input feature maps, E-CBAMs must be processed together with these maps. The intermediate activation maps’ spatial and channel-wise information has been preserved in the refined activation maps that result from the relationship with E-CBAMs. The overall quality of the feature representations is improved by the model’s ability to selectively pay attention to and emphasize important characteristics through this approach. The following Fig. 3 shows E-CBAM Architecture.

### Enhanced channel attention module

The primary goal of the enhanced channel attention module (ECAM) is to dynamically strengthen features that transmit important information while suppressing those that are less significant. The channel attention module uses global data to instruct the network’s architecture from the intermediate map and helps to recognize the significance of different channels in the feature maps from the intermediate features. The conventional CBAM block cannot identify edge information or the road border when lane markings have completely disappeared from the road or when a lane’s boundary is not visible. Sometimes there is a waste of computing resources and parameter build-up with the typical channel attention module. In this research, the MLP layer is eliminated from the channel attention module, and replaced with a 1D convolution layer to learn and localize significant channel-wise features immediately following average pooling. Subsequently, the outputs undergo processing using a sigmoid() and multiplicity of the intermediate feature to produce the channel-improved output features as shown in Eq. (2)

2 $${M}_{C}^{ECA}\left(I\right)=\sigma (1{D}_{k}\left({I}_{avg}^{C}\right))$$

where, *M*^*ECA*^ (*I*) is the C-th channel attention map, *σ* represents the sigmoid function, which maps its input to a value between 0 and 1. (1*D*_*k*_) stands for a 1D convolutional procedure with a k-sized kernel. The average-pooled feature map is subjected to this convolutional procedure, which captures patterns and relationships among channels and ${I}_{avg}^{c}$ indicates the average intermediate feature map from the channel part (C). Input for the Enchanced Spatial Attention Module (ESAM), a channel-refined feature is discussed below.

### Enhanced spatial attention module

The output of channel-refined features from the ECAM is sent to ESAM. The spatial dimension information of the input feature maps is focused and compressed using maxpool and avgpool in "Enhanced Spatial Attention Module". It then goes through a sharpening filter to enhance the information on edge features. Sobel Sharpening filters are used in the "Enhanced Spatial Attention" approach to increase spatial attention in the context of road lane detection. The ECAM of the CBAM is comparable to the ESAM module, which focuses on determining the optimum position and intensity of the road lane borders. The objective is to enhance the detection accuracy of road lane margin. Once the mapping feature channel is narrowed, the pooled output is subjected to a sharpening filter with weighted assignments. Further, the convolution layer reduces the feature map’s channel and passed through a sigmoid activation function. The product of the sigmoid function with channel refined feature is obtained to get the spatial refined feature maps. The process allows the system to make improvements and adjustments to the highway lane margin data. This is Mathematically presented in Eq. (3).

3 $${M}_{S}\left(I\right)= \sigma ({P}_{1X 1}\left({f}^{n X n}\left(\left[{I}_{avg}^{S};{I}_{max}^{S}\right]\right)\right))$$

where, $\upsigma$ signifies the sigmoid activation function, ${\text{f}}^{\text{n X n}}$ indicates sharpening filter, which is used to improve the concatenated feature maps’ spatial details with a filter dimension of n x n, and ${\text{P}}_{1\text{X }1}$ is a 1*1 pointwise convolution applied to the sharpened feature maps. ${\text{I}}_{\text{avg}}^{\text{S}}$ and ${\text{I}}_{\text{max}}^{\text{S}}$ is the average intermediate feature map and maximum intermediate feature map from the spatial part. ${I}_{avg}^{S}* {I}_{max}^{S}$ denotes the concatenation of the average-pooled and max-pooled feature maps along the channel dimension.The channel attention module’s average pooling operations make the process scale-invariant by capturing contextual information regardless of the amount of the input. Working with fixed-size pooling feature vectors, the 1D convolutional layer is flexible enough to function at any resolution. Because of its weight-sharing and local receptive fields, convolutional filters applied to spatial maps in the spatial attention module can accommodate variable spatial dimensions. Re-scaling element-wise multiplication enables CBAM to successfully improve feature representations at various scales and resolutions by matching attention maps with input feature maps regardless of resolution. Finally, the E-CBAM refined features and the last set of Stride convolution layers of features from the encoder module are then transferred to the decoder module will be discussed in the upcoming section.In this work, the 3 × 3 first order sobel sharpening to the utilization of the extracted features' edge data is reinforced.

## Decoder unit

The decoder unit in this architecture is a crucial component comprising concatenation, activation functions, de-convolutional blocks which are similar to encoder, and up-sampling layers. Each de-convolutional block consists of three consecutive conv2D transpose layers, all containing the same number of filters (1 × 1, 3 × 3 and 1 × 1). Following these convolutional layers, Attention normalization is applied, and three GELU activation functions are sequentially utilized to introduce non-linearity to the processed data. The process begins with the gathering of outputs from each E-CBAM block, including E-CBAM-1, E-CBAM-2, E-CBAM-3, and E-CBAM-4, which are combined with a 2 × 2 up-sampling layer in the decoder. The concatenation procedure specifically pairs outputs from E-CBAM-1 and E-CBAM-2, as well as E-CBAM-3 and E-CBAM-4, ensuring that no features are lost during this integration step. To construct two activation maps, the concatenated outputs are then carefully combined, emphasizing the effective integration of both low-level and high-level features. Notably, E-CBAM-2 and E-CBAM-4 undergo a 2 × 2 up-sampling process before concatenation to match the form of E-CBAM-1 and E-CBAM-3. Following this, three parallel de-convolutional blocks are employed, taking inputs of 2 × 2 up-sampling, E-CBAM-1, and E-CBAM-2. The necessity to recover the original features from each encoder block is the driving force behind the use of 2 × 2 up-sampling in this stage. The de-convolutional blocks further receive inputs up-sampled to 2 × 2, 4 × 4, and 16 × 16 to maintain consistency with the proportions of the original input images. This consistency ensures that the reconstructed features align with the initial input structure. Creating a single map that combines the minimal, excellent, and refined feature levels is the final phase. Concatenating all three feature maps yields this result. Next, a 1 × 1 convolution with a single filter is used to create a predicted image. The whole architecture is made to collect and aggregate data at several scales in order to provide a deeper and more accurate prediction.

## Experiments and results

Experiment to assess the results and robustness of the proposed framework was conducted and the evaluation metrics of the proposed model was analysed. Estimating lanes from road images in various scenarios such as damaged road, different weather conditions and curvy roads were recorded.

## Implementation details

In this work, three datasets are utilized, such as TuSimple (different weather conditions images), curve lanes (different curve lanes images) and cracks and potholes in road images (damaged road images), to train the proposed model. The suggested method has been trained to retain essential spatial information for accurate prediction.

**Figure 4:**
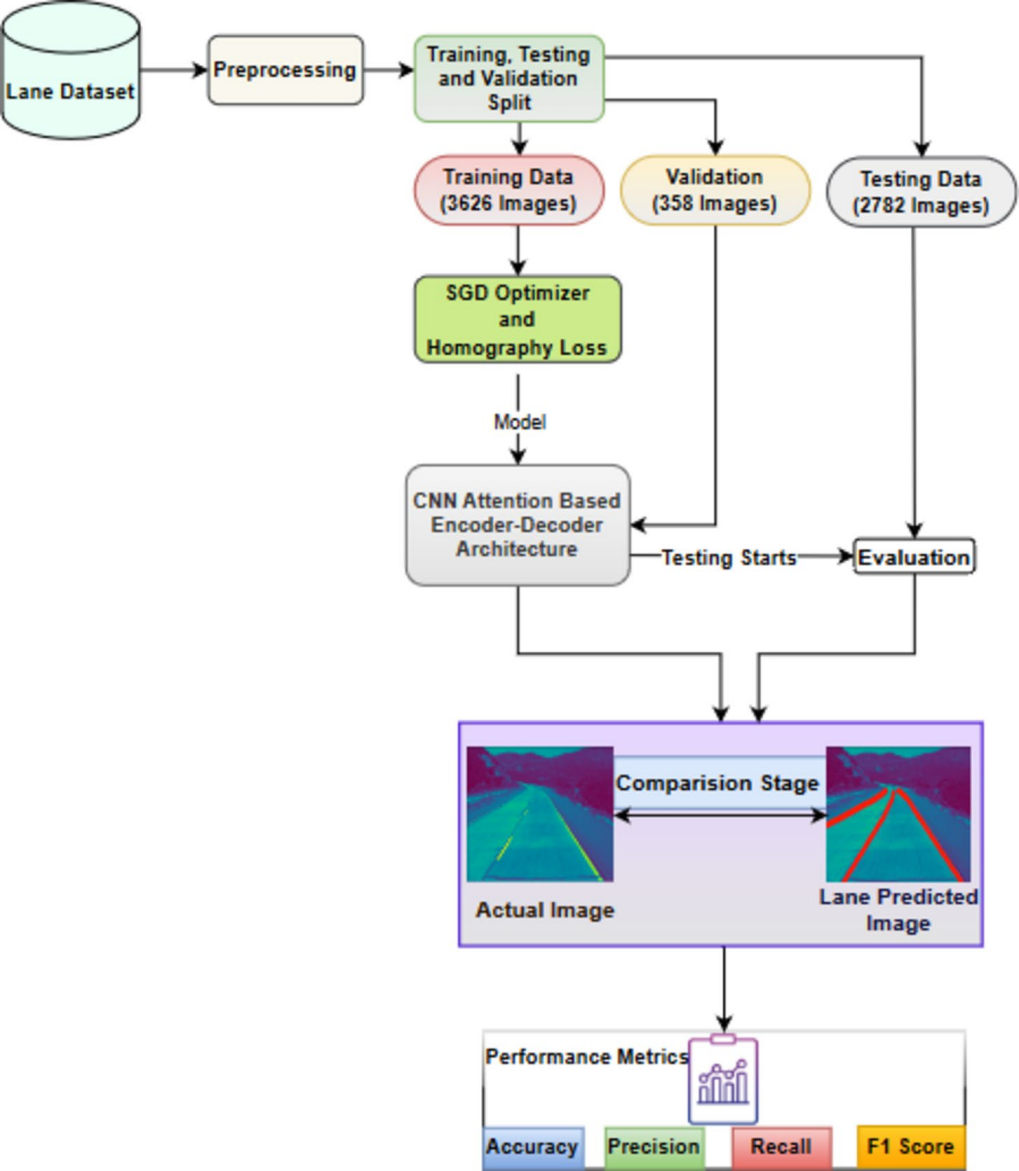
Overview of Evaluation Process.

Around 6,408 road images were obtained from TuSimple dataset on US highways^37^. The image has a resolution of 1280 × 720. From the available dataset, 3,626 images were used for for training, 358 images for validation, and 2,782 images for testing under different weather conditions. CurveLanes^38^is a brand-new benchmark lane detection dataset with 150 K lanes images for challenging traffic lane detection scenarios including curves and multi-lanes. It is gathered in actual urban and highway settings throughout many Chinese cities. It sets a more difficult bar for the community and is the largest lane detection dataset till date. We divided the entire 150 K dataset into three parts: 100 K for training, 20 K for valuation, and 30 K for testing. Most of the images in this dataset have a resolution of 2650 × 1440. Each image’s lanes are manually annotated with natural cubic splines and the selected images contain curvy lane. There are more challenging situations including S-curves, Y-lanes, night and multi-lanes in above dataset. The cracks and potholes in road images dataset^39^contain more than 5,000 images of potholes on actual roads taken in the wild. These photographs have a high resolution of 1920 × 1080 or above and were taken utilising their crowd sourcing platform from more than 2000 different places. From the available dataset 56 percentage is used for training and 44 percentage is used for testing on applying train-test-split technique from the scikit-learn package as shown in Fig. 4. The deep learning framework called Tensorflow version 2.12.0 and keras verison 2.12.0 is used in this work and deployed in Jupiter Notebook with Python 3.9 platform. Proposed model was trained using the Stochastic Gradient Descent (SGD) optimizer, a batch size of 10, a learning rate of 5e-5, and 50 epochs. The model was trained and experiments were carried out with Windows 11 pro, 32 GB of RAM and an Intel Core i9-12,900@ 3.20 GHz HP Z2 G9 SFF GPU processor.

The SGD Optimizer was chosen in this work to improve the performance of the adaptive learning rates and handle large datasets efficiently^40^. This optimizer speeds up the training process by updating model parameters based on a random selection of the training process which will reduce the processing time for the overall attention based encoder-decoder architecture^41^. The steps involved for SGD optimizer are shown below;

- Sort the training dataset in a random order.
- Split the dataset into smaller groups.
- Determine the gradient of the loss function with respect to
- the model parameters for every mini-batch.
- Scaled by a learning rate, update the model parameters in the gradient’s opposite direction.

## Performance results

Validating the efficiency of the mixed dataset and contrasting it with other standard methods is one way to assess its resilience and accuracy. As the goal of the proposed model is to distinguish between "Lane" and "Non-Lane" pixels, measures such as pixel accuracy, Dice-coefficient, Intersection over Union (IoU), precision, recall and F1 score was considered to evaluate it^42^. In the case of a binary segmentation task, pixel accuracy is defined as the percentage of properly categorised pixels. Pixel accuracy cannot be the greatest metric for evaluating the segmentation task, nevertheless, owing to the problem with class imbalance. Most of the Non-lane pixels in the dataset for lane recognition are highly out of balance in the images. As both of these are based on the area of overlapping among the real picture and the anticipated image, the Dice coefficient and the IoU are considered to be more effective metrics for segmenting the lane path to attain the best lane detection. Both metrics have significance for precise borders, shapes, and in assessing small objects in segmentation task because they are efficient in quantifying overlap, managing class imbalances, and being sensitive to partial matches. The Dice coefficient and IoU^43^ is derived from the Eq. (4) and (5) respectively.

4 $$Dice Coefficient=\frac{2\sum {x}_{predicted}{x}_{groundtruth}}{\sum {x}_{predicted}+{x}_{groundtruth}}$$

5 $$IoU=\frac{\sum {x}_{predicted}{x}_{groundtruth}}{\left(\sum {x}_{predicted}+\sum {x}_{groundtruth}\right)-\left(\sum {x}_{predicted}{x}_{groundtruth}\right)}$$

On dividing the two overlapping regions by the total number of pixels, it demonstrates the Dice coefficient and IoU simultane- ously shows the union region that separates the anticipated visuals from the ground truth.

**Figure 5:**
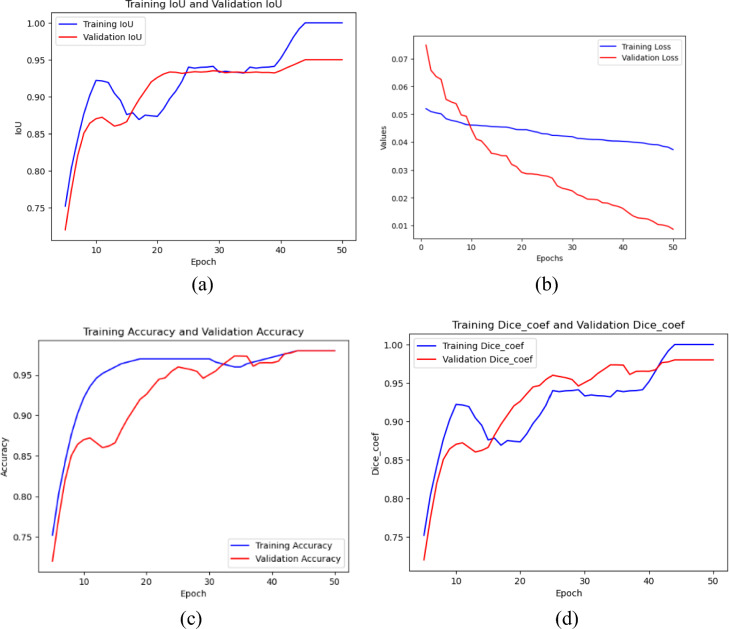
Curve of Loss, DC and IoU while Training and Validation at 50 epochs.

Figure 5a–d depicts the Loss, Dice coefficient, and IoU pattern of the proposed model over the epochs of both training and test datasets. From the first to about the fifth epoch, the Dice coefficient (Fig. 5d) and IoU curves (Fig. 5a) of the model started growing quickly. After the 20th epoch, as it grew it stabilised progressively.

However, the proposed model managed to account for this variance, and beginning with the subsequent epochs, the curves stabilised another time. In Fig. 5b,c, the accuracy starts to rise after the 15th epoch and remains stable throughout until final epochs. It is concluded from the loss graph that the train and test loss is maintained stable even after the increase in epochs.

**Figure 6:**
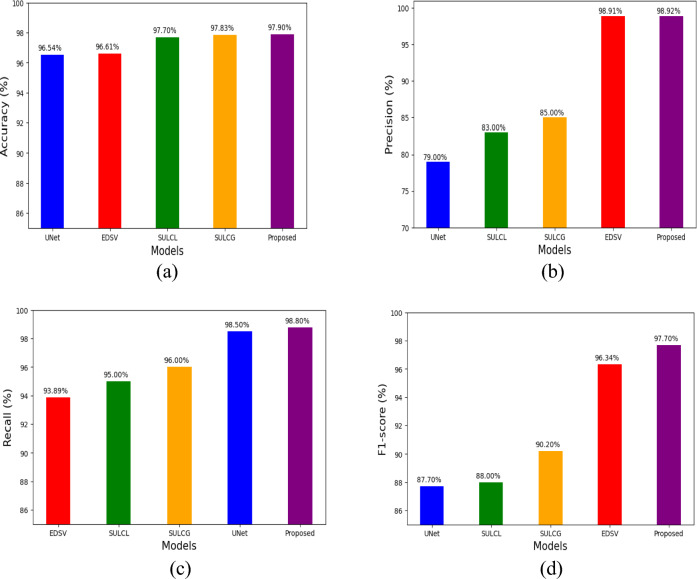
Comparison of various performance metrics of proposed with existing approaches.

Based on the parameters chosen for the test set, Figure 6a–d and Table 2 presents the results obtained with existing methods and the proposed method.Significant findings are shown by comparing the performance of several models, such as U-net, Encoder-decoder SegNet VGG16, SCNN Unet Light ConvGRU, SCNN Unet Light ConvLSTM, SHPDAN (Spatial Hierarchical Dilated Attention Network) and the proposed LD-CAM architecture. U-net achieves an F1 score of 87.7% while having a comparatively low accuracy of 79% and a high recall of 98.5%. This indicates that although U-net has a greater proportion of false positives, it is also strong at finding actual positives. The SegNet VGG16 encoder-decoder has a well-balanced performance, and excellent overall efficacy in maintaining low false positive and false negative rates, with accuracy of 96.61%, precision of 98.91%, recall of 93.89%, and F1 score of 96.3%.The F1 scores of 90.2% and 88%, respectively, for the SCNN Unet Light ConvGRU and SCNN Unet Light ConvLSTM models indicate that they perform better in terms of recall 96% and 95%, respectively, but less in terms of accuracy as 85% and 83%, when compared to the encoder-decoder SegNet VGG16 and SHPDAN has accuracy as 96.04%. Although these models show an excellent balance, they do not achieve peak performance. Nevertheless, with an accuracy of 97.90%, precision of 98.92%, recall of 98.8%, and an F1 score of 97.70%, the proposed LD-CAM architecture performs better than the other existing models. This results demonstrate LD-CAM’s sophisticated ability to precisely detect essential characteristics while reducing false positives and negatives. The model is the most efficient among those tested because of its high accuracy and recall, while it demonstrates how well it maintains a great balance. The results clearly present that the proposed approach performs superior compared to the methods on all measures. This suggests that proposed model will be more adaptable when used to create a real-world self-driving vehicle that recognises lanes in real-time to avoid accidents.

**Table 2:** Model performance comparison.

Model	Accuracy	Precision	Recall	F1 Score
U-net	96.25	79	98.5	87.7
Encoder-decoder SegNet VGG16	96.61	98.91	93.89	96.3
SCNN Unet Light ConvGRU	97.83	85	96	90.2
SCNN Unet Light ConvLSTM	97.7	83	95	88
SHPDAN	96.04	–	–	–
Proposed (LD-CAM architecture)	97.90	98.92	98.8	97.70

**Figure 7:**
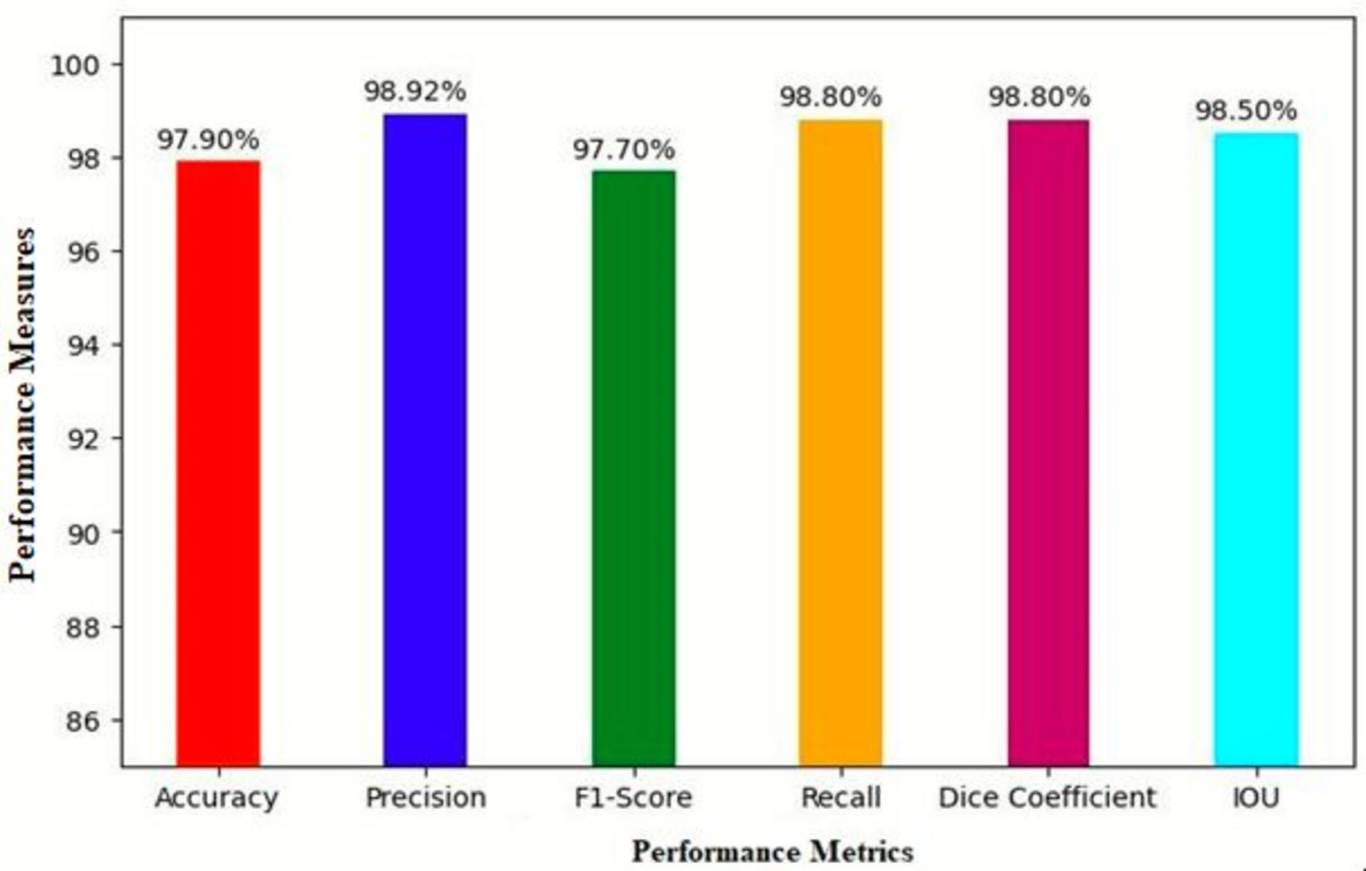
Performance metrics of the proposed method.

Figure 7 depicts the overall performances of the proposed method and the graph reveals that accuracy attained 97.90%, IoU of 98.50%, Precision of 98.92%, F1 Score of 97.70%, Recall of 98.80% and Dice coefficient as 98.80%.

Some visualisation results are provided that was obtained using the proposed approach to test the resilience of proposed model. The curvy road conditions are preferred for this experiment because properly detecting damage on a curved road is one amongst the most difficult tasks for existing algorithms.

**Figure 8:**
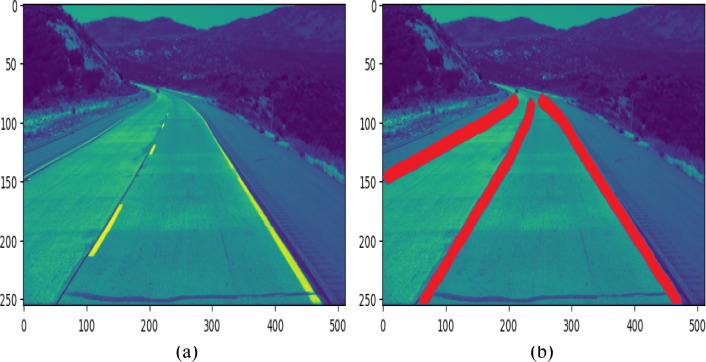
Lane detection in curve lane conditions.

Figure 8a,b depicts the original images and the associated anticipated images, with lanes highlighted in red at the curvy of the road stated below.

**Figure 9:**
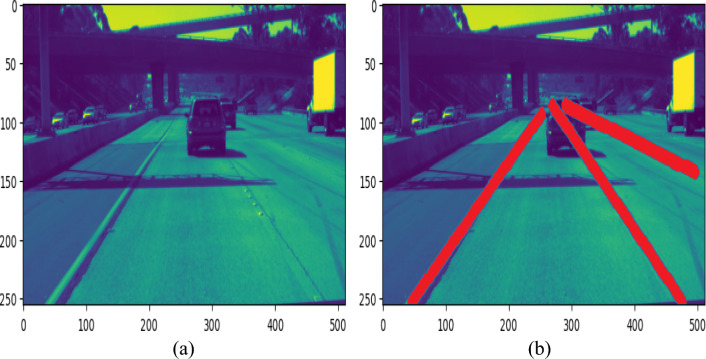
Lane detection in damaged road condition.

Poor pavement conditions are observed in many roads in developing countries. Also, roads are covered with huge holes and various cracks. Furthermore, the lane delineation is hazy and disappears from time to time. In this investigation, the aforementioned situation was analysed and the proposed model was challenged to anticipate lanes in such bad conditions. Figure 9a,b depicts the actual and projected frames by proposed model in the presence of faulty pavement conditions. The given images were employed to choose a route that had several openings and cracks in the roadways. The road has lost even its lane markers. However, the proposed algorithm accurately anticipated the image’s lane margins, which is excellent enough for a car to operate safely even in adverse conditions.

**Figure 10:**
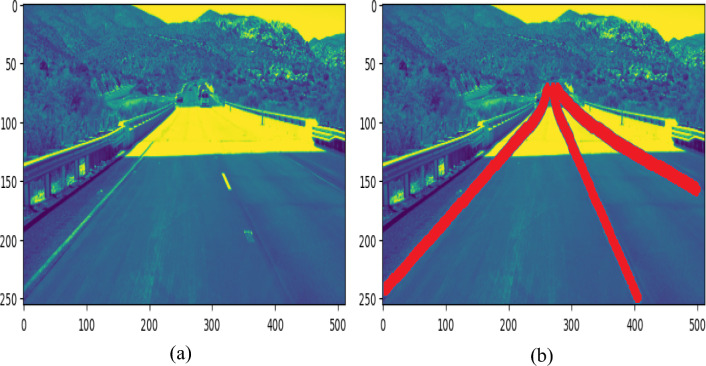
Lane detection in different weather conditions.

Stochastic Gradient Descent (SGD) optimizer, and Gaussian Error Linear Unit (GeLU) activation function was applied. The lanes on these roadways are adequately delineated, and the roadway is in good shape. For this experiment, ideal weather and lighting conditions are chosen and an experiment was carried out is shown in Fig. 10a,b. Figures 8a and 10b shows that the proposed model estimated the lanes effectively in all circumstances. As a result, when compared to the results of other models, the proposed framework predicted the lane and performed better in terms of accuracy, Dice loss, Dice coefficient, and IoU.

## Conclusion

In this article, a CNN based encoder-decoder model is used for precise lane detection based on attention mechanism. The proposed strategy achieved effective and accurate lane recognition especially under challenging circumstances because of the accurate data collected by the attention modules. The evaluation showed that the proposed approach could achieve greater Dice Score, Accuracy, IoU, Precision, F1 score, and Recall when compared to the existing methods. Additionally, the recommended strategy demonstrated its robustness in a variety of difficult settings by producing great qualitative results. Although this technique may be used on both structured and unstructured roads, it performs well on unstructured roads when there are significant damages to the road and no lane markers are available in various weather situations. Though the LD-CAM model shows promising results its computational complexity may increase due to introduction of enhanced convolutional attention mechanism (E-CBAM), while deployed in low power embedded systems commonly used in autonomous vehicles. Future work will focus on optimizing the E-CBAM to reduce computational overhead while maintaining high detection accuracy. Additionally, although the model performs well in extreme weather conditions, its performance under varying lighting conditions, such as sudden transitions from sunlight to shadows, needs further investigation. Incorporating adaptive illumination techniques or additional preprocessing steps could enhance the model’s robustness in these scenarios.Expanding the training dataset to include a broader range of road types and conducting extensive field tests will improve the model’s generalization. Furthermore, the model’s accuracy heavily relies on the quality of the input data. Low-resolution or noisy images can degrade its performance. Developing advanced noise reduction techniques and enhancing the model’s ability to work with low-quality data are essential areas for future research. By addressing these limitations, we aim to further enhance the robustness and applicability of the proposed LD-CAM model in real-world autonomous driving scenarios.

## Data Availability

The data that support the findings of this study are available from the author, upon reasonable request.
